# NREM delta power and AD-relevant tauopathy are associated with shared cortical gene networks

**DOI:** 10.1038/s41598-021-86255-6

**Published:** 2021-04-08

**Authors:** Joseph R. Scarpa, Peng Jiang, Vance D. Gao, Martha H. Vitaterna, Fred W. Turek, Andrew Kasarskis

**Affiliations:** 1 Department of Anesthesiology, Weill Cornell Medicine, New York, NY 10065 USA; 2 Center for Sleep and Circadian Biology, Department of Neurobiology, Northwestern University, Evanston, IL 60208 USA; 3 Icahn Institute for Genomics and Multiscale Biology, Department of Genetics and Genomic Sciences, Icahn School of Medicine at Mount Sinai, New York, NY 10029 USA; 4 Department of Population Health Science and Policy, Icahn School of Medicine at Mount Sinai, New York, NY 10029 USA

**Keywords:** Circadian rhythms and sleep, Molecular neuroscience, Network models, Alzheimer's disease

## Abstract

Reduced NREM sleep in humans is associated with AD neuropathology. Recent work has demonstrated a reduction in NREM sleep in preclinical AD, pointing to its potential utility as an early marker of dementia. We test the hypothesis that reduced NREM delta power and increased tauopathy are associated with shared underlying cortical molecular networks in preclinical AD. We integrate multi-omics data from two extensive public resources, a human Alzheimer’s disease cohort from the Mount Sinai Brain Bank (N = 125) reflecting AD progression and a (C57BL/6J × 129S1/SvImJ) F2 mouse population in which NREM delta power was measured (N = 98). Two cortical gene networks, including a CLOCK-dependent circadian network, are associated with NREM delta power and AD tauopathy progression. These networks were validated in independent mouse and human cohorts. Identifying gene networks related to preclinical AD elucidate possible mechanisms associated with the early disease phase and potential targets to alter the disease course.

## Introduction

Alzheimer’s disease (AD) emerges over the course of decades, involving a dynamic integration of genetic, environmental, and behavioral risk. A growing body of literature has elucidated the neurobiological changes in the preclinical phase of AD, noting significant neuropathologic and behavioral changes before the onset of cognitive decline. Studies of patients with dominantly inherited Alzheimer’s disease have demonstrated that amyloid-beta and tau accumulate for many years before pathologic changes in memory and executive function^1,2^, and Aβ levels peak around the time clinical symptoms manifest^3^. These early stages of AD also are accompanied by widespread multi-region loss in synaptic density and neuronal populations^3^. This neuropathologic evidence complements the data demonstrating a complex neuropsychiatric symptomology in the early stages of AD^4–7^. Recent studies have shown that the preclinical phase includes anxiety-depressive symptoms^8^ and increased loneliness^9,10^. Other work has noted significant changes to sleep–wake cycle and its influence on AD pathology^11–21^. This body of literature is highly concordant with those of Parkinson’s disease and Huntington’s disease that demonstrate pathologic and behavioral changes during the prodrome^22–28^. These characteristics may indicate a generalizable feature of neurodegenerative disease and may point to opportunities for early intervention.

Sleep and circadian disruption is a significant cause of morbidity in AD. This stressor is transmitted through the patient’s social and familial network, affecting the mental health of families and caretakers^29–32^. Often sleep and circadian disruption leads to the institutionalization of patients in long-term care facilities that more reliably offer 24-h care. Though sleep disruption plays a significant role in late-stage AD, the relationship between sleep and dementia is highly intertwined throughout the progression of Alzheimer’s disease^16,18–20,33^. Epidemiologic evidence indicates that sleep deprivation may increase dementia risk, and dementia often leads to sleep deprivation, potentially creating a feedback loop^13–16,18,20,33,34^. A number of sleep changes have been associated with AD pathogenesis^11,14,15,35–37^. Recent work has demonstrated a reduction in NREM sleep in preclinical AD, pointing to its potential utility as an early marker of dementia^35^. Reduced NREM in humans is associated with increased atrophy and Aβ accumulation in the medial prefrontal cortex and tau accumulation in specific Brodmann areas in the frontal, temporal, and parietal brain regions^35,38–40^. This work shows particular sleep features that manifest in the preclinical phase and are associated with specific elements of AD pathology, but the molecular mechanisms underlying these neurophysiologic markers are poorly understood. Understanding the biological basis of these complex features of preclinical AD may reveal insights into pathogenesis of preclinical Alzheimer’s and novel opportunities for therapy.

The motivation of the present work is to corroborate the relationship between NREM slow-wave activity (SWA) and cortical tau pathology at the molecular level and characterize the shared molecular basis of these intertwined phenotypes^35^. We use data from two extensive public resources, a human Alzheimer’s disease cohort from the Mount Sinai Brain Bank (N = 125) reflecting AD progression^41^ and a (C57BL/6J × 129S1/SvImJ) F2 mouse population in which NREM delta power was measured (N = 98)^42^, to test the hypothesis that specific gene networks in the prefrontal cortex are associated with both NREM sleep and AD-related tau pathology. These large-scale studies catalogue extensive genetic and molecular information across species—rich public resources that can be used to investigate specific biological questions not considered in the original studies. By studying genetic, environmental, and phenotype data across species, we can characterize how genetic variation and gene expression integrate information in gene networks and identify molecular pathways robustly associated with both NREM sleep and tau development.

## Methods

### Multiscale data generation in mouse and human cohorts

We previously described in full detail the experimental design, quality control, and measurements of genetic, gene expression, and phenotypes in ninety eight (98) members of (C57BL/6J × 129S1/SvImJ) F2 mouse population^42^. We used these mouse data for our analyses in the present study, and all animal experiments that generated these data were approved in advance by the Institutional Animal Care and Use Committee at Northwestern University and were in compliance with the Federal Animal Welfare guideline. Briefly, two hundred and eighty three (283) behavioral, affective, and electroencephalographic traits were measured, including NREM delta power. Mice starting at 7 weeks of age were subjected to a battery of behavioral test and at 10 weeks of age were surgically implanted with electroencephalogram (EEG) and electromyogram (EMG) electrodes for sleep/wake recordings. Two stainless steel screws (Small Parts, Miami Lakes, FL) serving as the EEG leads were screwed into the skull and placed in the cerebral cortex with one screw located 1 mm anterior to bregma and 2 mm lateral to the central suture (somatomotor areas), and the other (i.e., the reference electrode) at 1 mm anterior to lambda and 2.5 mm lateral to the central suture (visual areas). As is typical for studies in mice, one-channel EEG signals were used here as a proxy to describe the characteristics of cortical frequency spectrum in an individual animal. Two weeks after the surgery, three episode of continuous EEG/EMG recordings were made, including (1) a 24-h undisrupted baseline (ZT0 to ZT0; ZT0 = light on), (2) 6 h of sleep deprivation (starting at ZT2; i.e., 2 h after the end of baseline) followed by 16 h recovery period, and (3) sleep/wake after 1 h (ZT5–ZT6) of restraint stress given a week after the sleep deprivation. EEG/EMG recordings were manually scored in 10-s epochs into NREM, REM, and wake, and then quantified into sleep phenotypes. For our current analysis, we focused on the relative NREM delta power during the 24 h baseline sleep, the phenotype referred to as “BL.24h.nrem.delta” throughout the original manuscript. The power spectrum analysis was done epoch-by-epoch. Raw power of the delta band (1–4 Hz) was averaged for all NREM epochs in the 24-h baseline and was then expressed as a proportion of the epoch-averaged NREM total power of the 1–30 Hz range during the same period. The normalization to total power removes the variations in the EEG signal amplitude among individual animals, which are largely technical rather than biological variations. Our phenotype of interest, the relative NREM delta power during baseline reflects, the intensity of NREM delta activities, which is a commonly used marker of sleep homeostatic drive and is highly heritable^43–45^. A closely related phenotype, NREM delta energy, which is a function of delta power and the amount of slow-wave sleep, is not analyzed here or in our previous study. Finally, as animal cohorts were found to have a widespread impact on the 283 phenotypes measured in this mouse population (ANOVA P values significantly deviate from a uniform distribution and are distorted toward smaller values), linear adjustments were used to remove cohort effects for all phenotypes. For each phenotype, phenotypic values of each animal were fitted in a linear regression model with cohort as the independent variable, and intercept + residuals were taken as the adjusted values. This linear procedure was taken because it is conceptually simple and compatible with subsequent analyses (e.g., correlations) which are also linear.

All mice were genotyped using the Affymetrix MegAllele genotyping mouse 5K SNP Panel, of which 2458 were polymorphic between the C57BL/6J and 129/SvImJ inbred strains. To measure gene expression, Affymetrix GeneChip Mouse Genome 430 2.0 Array was used. Mice were left undisturbed for two weeks before euthanasia and tissue collection. All dissections were performed between ZT6 and ZT7, and four brain regions were collected, including frontal cortex (cortical tissue rostral to the striatum), hippocampus, thalamus, and hypothalamus. Only the expression data in the cortical tissue were used the current study. Raw data were normalized using robust multiarray averaging with quantile normalization and consequently log_2_-transformed. We adjusted data for effects of technical covariates, including RNA processing batch, array batch, and animal cohorts. Principal components analysis was used to capture the unknown source of variations, and data were further adjusted by treating principal components as covariates if they were not associated with any genomic loci [logarithm of the odds (LOD) < 3]. Lastly, multiple probesets mapping to a single gene were reduced to the gene level by calculating their median. The bottom 10% of genes by variance were removed to reduce noise in calculating gene coexpression, and samples with an interarray correlation two standard deviations away from the mean were considered outliers and removed. Gene expression and phenotype data are publicly available at GSE109112.

Data from the Alzheimer’s disease cohort was collected by the Mount Sinai Brain Bank, and full description of the experimental methods and data generation procedures have been previously published^41^. These data include 1053 postmortem brain tissues from 125 human brains. Gene expression was measured and merged from two Affymetrix microarray platforms, Human Genome U133A and U133B. Probes measured on both platforms were averaged before the gene expression matrix was corrected for covariates using linear regression. For each member of the cohort, clinical dementia rating (CDR), diagnostic certainty score through the Consortium to Establish a Registry for Alzheimer’s disease (CERAD), Braak Score, and estimates of neuritic plaque (NPL) and neurofibrillary tangle (NFT) density were measured using a previously published protocol^46^. This cohort includes individuals expressing the full spectrum of clinical and histopathologic phenotypes, from normal to severe, in order to correlate variations in gene network expression with clinical and histopathological status and disease progression. Gene expression data is publicly available at GSE84422, and all data can be accessed through Synapse.

### Calculating coexpression and module enrichment to identify functional molecular relationships

Both previous studies which we primarily examine in this work use weighted gene coexpression network analysis (WGCNA) to calculate gene coexpression associations in high-dimensional microarray data^41,42^. We further analyzed these coexpression networks to identify molecular relationships between tauopathy and non-REM slow wave activity. These coexpression networks were previously generated using a similar generalized methodology. For each cohort, the correlation matrix was calculated between all genes. This correlation matrix was then raised to a power, ß, to calculate the adjacency matrix^47^, which was quadratically transformed to the topological overlap matrix to capture higher-level gene–gene relationships, like nearest neighbor associations^48^. Hierarchical clustering revealed gene coexpression modules, which were assigned arbitrary colors for identification. Each color represents a different module, which was catalogued in the original manuscript. The first principal component of each module (module eigengene) was correlated with relevant phenotypes to determine associations between modules and traits. In the original F2 mouse study, twenty-six coexpression modules were identified. Notably, gene expression in three modules (“blue”, “green”, and “skyblue”) were found to be altered both by sleep deprivation and major depression. Network images were generated using Cytoscape v3.7.2^49^.

To identify gene networks associated with both AD-relevant tauopathy and non-REM slow wave activity, we compared module memberships between all previously calculated coexpression modules in the frontal cortex of the (C57BL/6J × 129S1/SvImJ) F2 mouse population and BA10 of the human AD cohort. Fisher’s exact test was used to calculate module overlap and a module was considered conserved when Bonferroni-corrected p-value < 0.05 and Odds Ratio > 2. Fisher’s exact test was also used to investigate whether the two conserved modules were overrepresented with genes differentially expressed in non-demented individuals with AD histopathology when compared to an age- and sex-matched cohort that was clinically and histopathologically normal. Nominal p-values were reported since only two modules were investigated. Functional characterization of modules were calculated using DAVID^50,51^, and Benjamini–Hochberg p-values are reported. Furthermore, enrichment using ChIP-seq and CLOCK-knockdown data was performed with Enrichr and corrected p-values were reported^52^. The ChIP-seq data was catalogued in the ChEA database, derived from an experiment examining CLOCK-binding targets in 293T cells^53^. The knockdown data is publicly available from GSE50588, generated in GM19238 cell lines after siRNA knockdown, and catalogued in Enrichr under “TF Perturbations Followed By Expression”. Lastly, Fisher’s exact test was used to test two networks for enrichment for causal cognition genes. The cognition gene list was assembled by querying the Mouse Genome Database^54^ under the general category, MP:0002063. Next, genes in the two subcategories, enhanced learning (MP:0012314) and enhanced conditioning behavior (MP:0012316), were removed to ensure that the final gene list reflected genes causal solely for decreased cognitive behaviors. Fisher’s nominal p-values were reported since only two modules were tested. Odds ratios for all modules were plotted for the sake of comprehensiveness.

### Probabilistic causal modeling to identify directed gene–gene relationships

In this analysis, we examined previously reported graphical models representing gene–gene relationships in the F2 mouse population^42^. Constructing probabilistic causal models of gene expression data have been extensively detailed elsewhere^55^. Briefly, these probabilistic causal models are directed acyclic graphs in which each node represents gene expression of a gene, and the relationship between genes is captured by the conditional probability between nodes^56^. Monte Carlo Markov Chain (MCMC) simulation was used to reconstruct one thousand gene networks that fit the gene expression data and the fit of each reconstruction was assessed with Bayesian Information Criterion (BIC)—a metric conservative to overfitting since it gives a lower prior probability to more complex models (i.e. with more parameters). A consensus network was consequently calculated by keeping edges that appear in more than 30% of the 1000 reconstructions to balance sensitivity and specificity^57^. To infer causality in Markov equivalent structures, we used previously calculated cis-eQTLs as priors^42^ and prevented genes without cis-eQTLs from being parents of genes with cis-eQTLs, under the assumption that they are primarily regulated by genetic variation.

### Calculating key drivers of gene networks from probabilistic causal models

To calculate key drivers, we used a variant of module enrichment that utilizes the directedness of the probabilistic causal graphs^55,58^. For each module of interest, the downstream neighborhood of each gene was calculated, and nodes whose downstream network is greater than two standard deviations above the mean are considered network drivers. This method classifies nodes based upon their downstream network and uses the principle that nodes with more downstream nodes likely have a greater influence on the gene expression of the module as a whole. Consequently, key drivers are those nodes with significantly larger downstream networks than the average node in the module.

## Results

### Characterizing functional gene networks associated with the NREM delta power

**Figure 1:**
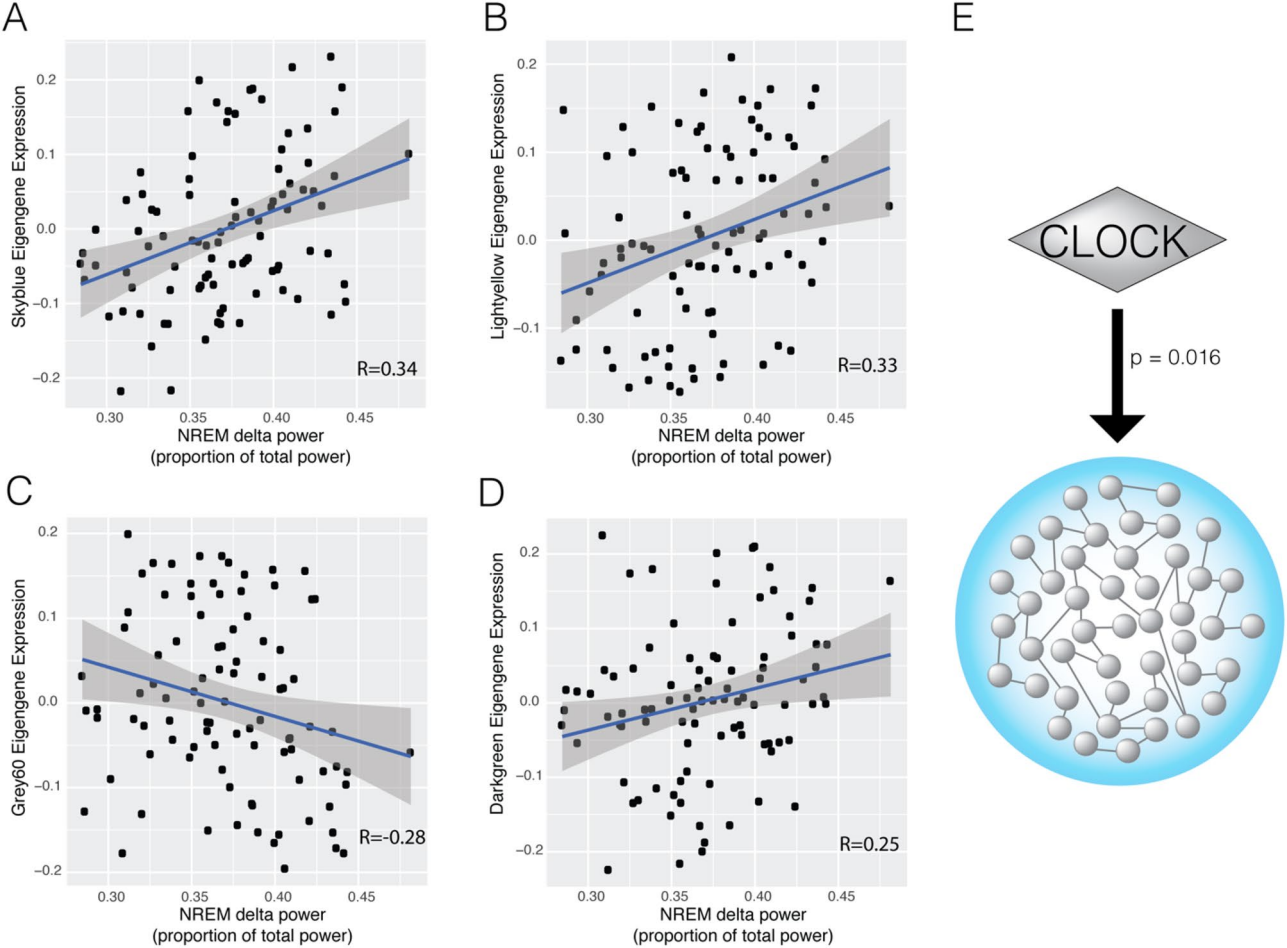
NREM delta power correlates with module eigengene expression of skyblue (**A**), lightyellow (**B**), grey60 (**C**), and darkgreen (**D**) modules. Correlation coefficients for each module-trait relationship is denoted in its respective panel. CLOCK knockdown directly modulates the expression of the skyblue module, with Fisher’s p-value reported in the figure (**E**). Summary statistics for panels (**A**–**D**) were originally represented in Supplementary Table 3 in PMID: 30050989.

In a previous study, two hundred eighty-three (283) behavioral, affective, and neurophysiologic phenotypes were measured in a population of (C57BL/6J × 129S1/SvImJ) F2 mice, revealing gene network pleiotropy and novel relationships between sleep–wake and behavioral traits^42^. We used these data to specifically investigate cortical gene networks associated with NREM delta power. Notably, these gene networks were reconstructed in cortical regions rostral to the areas where the EEG leads were placed. Although the regulation of NREM delta power and NREM homeostasis has a strong local component^59,60^, it also involves many brain areas. Particularly, prefrontal cortex in both humans and mice plays an important role in regulating NREM delta waves recorded in other cortical regions^39,61^. Four cortical gene networks were correlated with NREM delta power during baseline conditions (Fig. 1A–D): darkgreen (r_s_ = 0.25, P = 0.02, FWER = 0.03), grey60 (r_s_ = − 0.28, P = 0.006, FWER = 0.01), lightyellow (r_s_ = 0.33, P = 0.001, FWER = 0.005), and skyblue (r_s_ = 0.34, P = 7.6 × 10^–4^, FWER = 0.003). Skyblue showed the strongest association with NREM delta power and was previously implicated in both sleep deprivation and major depressive disorder^42^. This network includes circadian clock genes and clock-regulator genes *Per1*, *Per2*, *Bhlhe40*, and *Klf10*. Further analyses of ChIP-seq and gene expression data demonstrated that skyblue genes are targeted by CLOCK (P = 9.2 × 10^–10^, Q = 7.6 × 10^–8^) and their expression is strongly modulated by CLOCK knockdown (P = 1.58 × 10^–3^, Q = 0.016)^62^ (Fig. 1E). These results indicate that NREM delta power is correlated with four cortical gene networks and most strongly associated with the expression of a CLOCK-dependent cortical gene network.

### Identifying NREM delta power gene networks associated with AD progression and tauopathy

**Figure 2:**
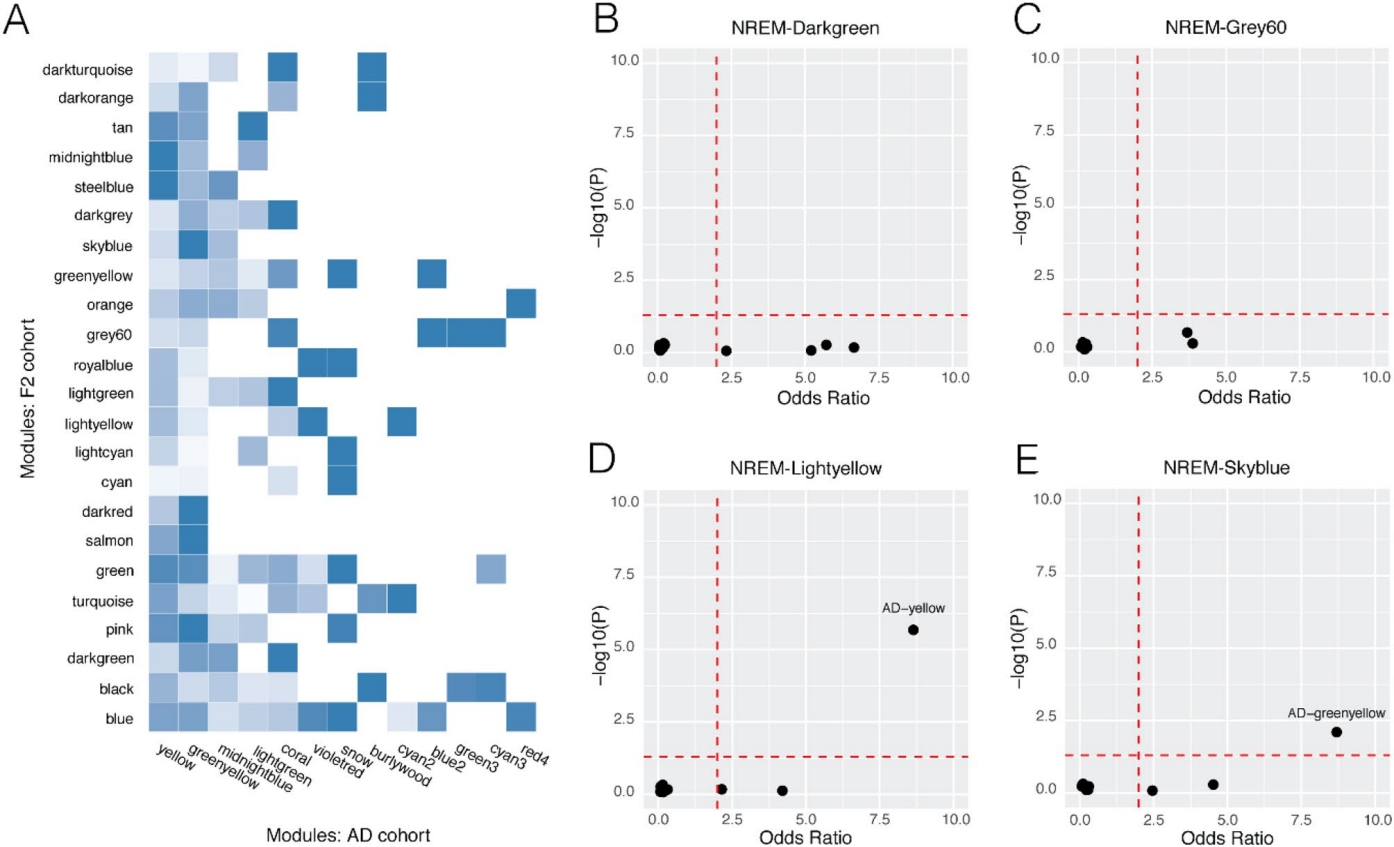
Sleep-relevant gene networks were compared directly with AD-relevant gene networks to determine network conservation (**A**). The darkness of the blue is proportional to the Fisher’s exact test odds ratio, with darker colors suggesting greater overrepresentation. Each NREM network enrichment is represented on an independent plot (**B**–**E**). NREM network enrichment for each AD-relevant network is represented by an individual point on the plot. The odds ratio is plotted on the x-axis and the − log10 of the Fisher’s P value plotted along the y-axis. (**B**–**E**) NREM networks were considered conserved in the AD cohort when odds ratio > 2 and P < 0.05, represented by the dotted red lines respectively.

Reduced NREM slow wave activity was associated with AV-1451 tau levels in PET scans of Brodmann area (BA) 10^35^. Previous experiments have measured histopathologic markers and BA10 gene expression in patients with clinical symptoms across the AD spectrum, including patients with no symptoms at all^41^. These experiments indirectly captured information regarding the development of AD pathogenesis and the gene networks involved. We reasoned that the association between human NREM slow wave activity and AD tauopathy may be reflected by shared underlying gene networks. To investigate this relationship, we compared gene networks associated with NREM delta power to those networks previously associated with AD progression. This analysis showed that sixteen of the twenty-three (70%) cortical modules in the F2 mouse population are conserved in the AD cohort. Of the modules associated with NREM delta power, lightyellow and skyblue are overrepresented in at least one AD gene network (Fig. 2A–E).

Next, we investigated if the AD modules associated with NREM delta power were also correlated with levels of tauopathy. NREM-lightyellow was overrepresented in AD-yellow (P = 2.7 × 10^–6^, OR = 8.5 [4.1–16.8]), a gene network previously correlated with Braak score (r = − 0.29, P = 0.022) and neurofibrillary tangle density (r = − 0.33, P = 0.008). NREM-skyblue was overrepresented in AD-greenyellow (P = 0.01, OR = 8.5 [2.9–21]), which was primarily associated neurofibrillary tangle density (R = − 0.25, P = 0.046). Both AD-yellow and AD-greenyellow are strongly enriched for known Alzheimer’s disease genes (P = 1.8 × 10^–4^ and P = 1.1 × 10^–3^, respectively), further confirming their relevance to AD pathogenesis. AD-yellow is composed of genes primarily related to synaptic function (P = 1.6 × 10^–21^), long-term potentiation (P = 8.8 × 10^–5^), and circadian entrainment (P = 2.7 × 10^–3^), while AD-greenyellow is primarily associated with mitochondrial biology (P = 1.9 × 10^–14^) and metal ion binding (P = 5.3 × 10^–3^). These results indicate that two cortical gene networks are associated with NREM delta power and AD tauopathy.

### Gene networks associated with delta power are modulated in non-demented individuals with neuropathological evidence of Alzheimer’s disease

**Figure 3:**
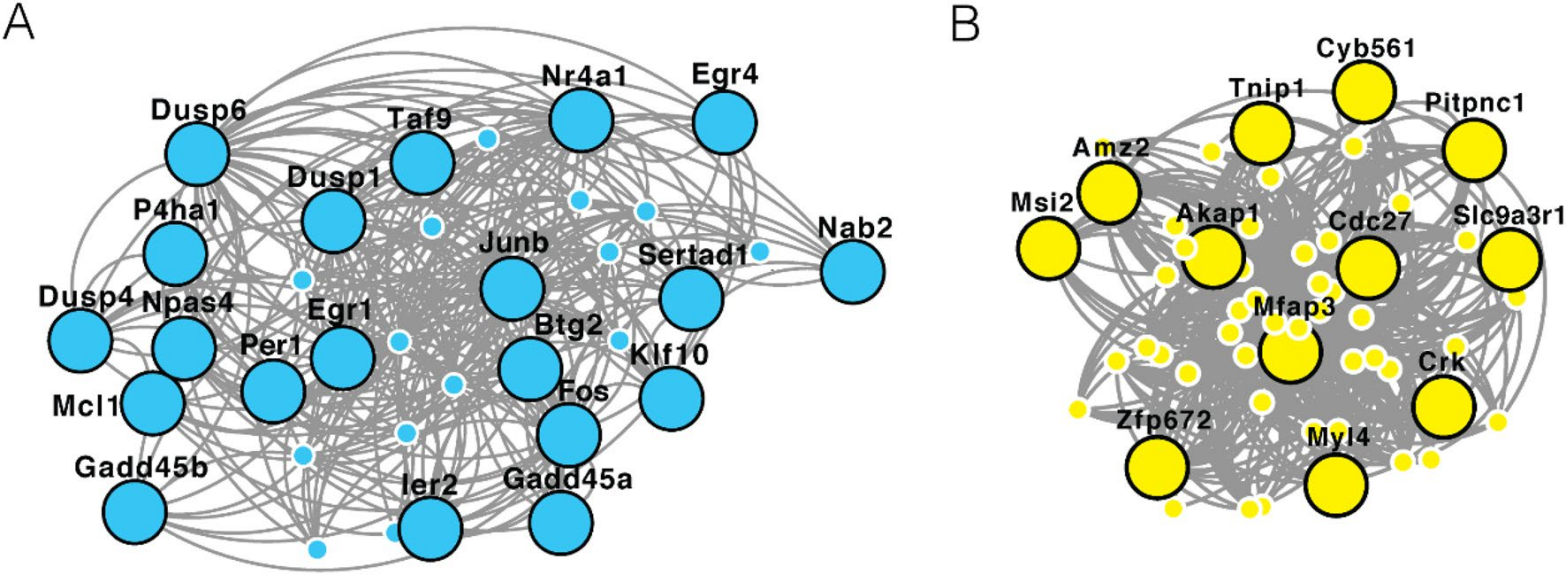
Coexpression networks for skyblue (**A**) and lightyellow (**B**) were calculated using weighted gene coexpression network analysis. The large, labeled nodes are the module members that are modulated in non-demented individuals with Alzheimer’s neuropathology. Network images generated using Cytoscape v3.7.2 (https://cytoscape.org/).

Gene networks relevant to NREM delta power and Alzheimer’s-related tauopathy may indicate aspects of the molecular pathogenesis evident in the early stages of Alzheimer’s disease. We have provided indirect evidence of this by identifying NREM delta power gene networks relevant to AD progression. To validate this finding, we examined an independent cohort of non-demented individuals with evidence of Alzheimer’s neuropathology (NDAD)^63^. These NDAD data were generated from BA10 and BA11 using laser capture microdissection and compared to an age- and sex-matched cohort that was both histopathologically and clinically normal. These data provide a useful approximation for examining the relevance of NREM delta power gene networks in preclinical AD. We reasoned that the two NREM delta power gene networks would be strongly modulated in NDAD if these networks reflected the early molecular stage of Alzheimer’s disease. Our analysis showed that genes differentially expressed in NDAD compared to their histopathologically and clinically normal controls (Benjamini–Hochberg FDR < 0.1) are overrepresented in NREM-lightyellow and NREM-skyblue. NDAD strongly modulates both NREM-lightyellow (P = 0.005, Odds Ratio = 3.6 [1.4–9.3]) and NREM-skyblue (P = 3.4 × 10^–7^, Odds Ratio = 7.5 [3.2–19.6]), differentially regulating approximately 22% of the NREM-lightyellow gene network and 59% of NREM-skyblue (Fig. 3).

### Integrative probabilistic causal models reveal gene network regulators associated with delta power and preclinical AD

**Figure 4:**
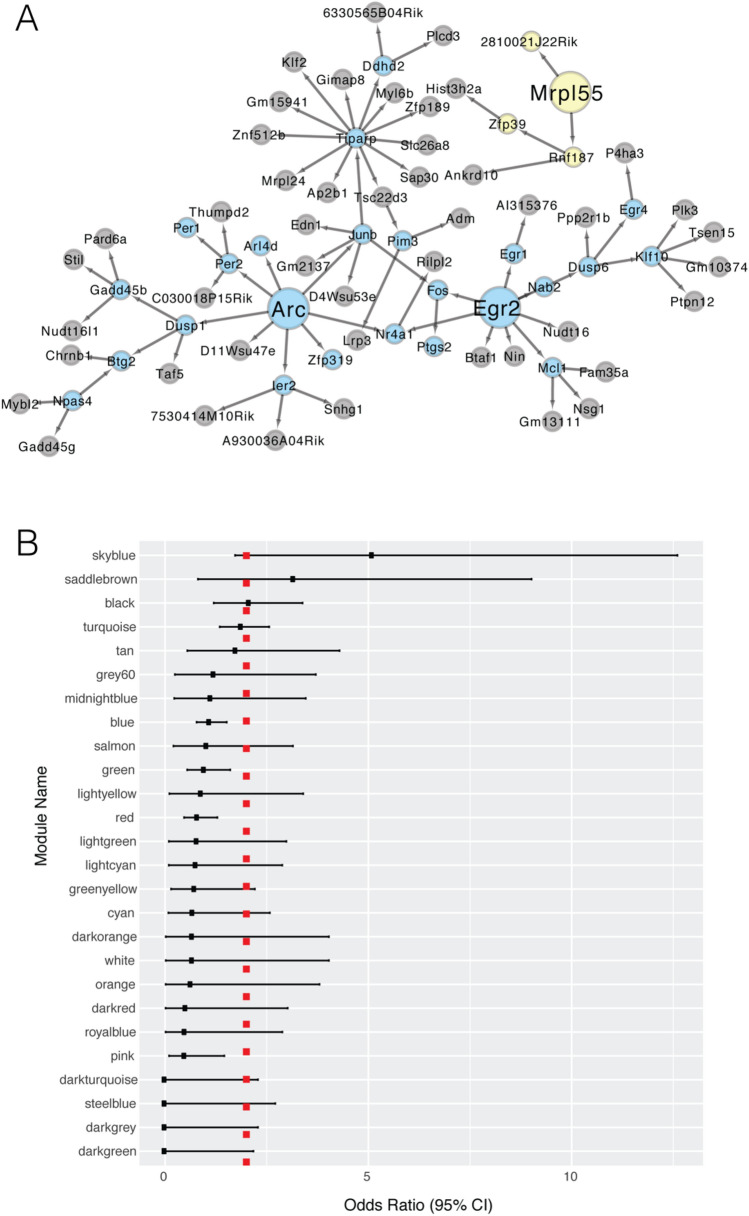
These direct acyclic graphs represent a subset of the transcriptome-wide gene–gene relationships involving skyblue and lightyellow genes (**A**). Skyblue and lightyellow nodes are highlighted by their respective colors, while all other genes, irrespective of their modules, are pictured in grey. The larger nodes represent the key regulators. Our panel (**A**) was generated using Cytoscape v3.7.2 (https://cytoscape.org/) and depicts a Bayesian network that was partially reproduced in Fig. 6B of PMID: 30050989. The Fisher’s odds ratio and 95% confidence interval are pictured (**B**), representing each network’s enrichment for genes known to cause abnormalities in learning, memory, and executive function. Networks are sorted from highest to lowest odds ratio. Dotted red line represents an odds ratio of 2.

Probabilistic causal models offer an effective strategy to integrate genetic variant and gene expression data and predict directional relationships between genes^57^. These models identify highly reproducible gene–gene relationships^64^, and empirical validation has demonstrated that these methods can successfully identify causal regulators in gene networks across a variety of species^57,65–67^, including humans with late-onset Alzheimer’s disease^58,68,69^. Given the phenotypic and transcriptional relationship between NREM and AD, we reasoned that key drivers of NREM-skyblue and NREM-lightyellow may contribute to preclinical AD pathogenesis and AD progression. Previous work investigated the intramodular and intermodular gene–gene regulation in this F2 mouse population^42^. This earlier analysis independently highlighted the gene–gene relationships and key drivers in one of our networks-of-interest, NREM-skyblue, because it is strongly modulated by sleep deprivation and major depression. *Arc* and *Egr2* were identified as key regulators of NREM-skyblue (Fig. 4A) and intermodular analysis demonstrated that it was upstream of a number of other functional pathways, including genes involved in respiratory transport chain, TCA cycle, mitochondrial function, and synaptic processing^42^. In the present analysis, we also specifically report the gene–gene regulatory network of NREM-lightyellow. Analysis of the directed gene–gene network in NREM-lightyellow revealed that *Mrpl55*, a mitochondrial ribosomal protein, was its primary regulator, upstream of genes altered in NDAD (Fig. 4A). These key drivers highlight the importance in preclinical AD of core components of bioenergetics and synaptic processing.

### Examining NREM delta power networks for known causal genes in learning, memory, and conditioning

Lastly, we reasoned that these networks may causally influence cognitive behaviors. We projected genes known to cause abnormalities in learning, memory, and conditioning in mouse models^54^ onto NREM-lightyellow and NREM-skyblue to determine if these networks may play a causal role in cognition. NREM-skyblue is strongly overrepresented with genes known to alter cognitive traits [P = 0.002, OR = 5.1 (1.7–12.6)] (Fig. 4B). These genes include the subnetwork key regulator, *Arc,* but known cognition-related genes are found throughout the downstream networks of both *Arc* and *Erg2*. On the other hand, we did not find such causal evidence for NREM-lightyellow [P > 0.05, OR = 0.89 (0.11–3.4)] (Fig. 4B), implying that its role in NDAD is not strongly causal or not captured by existing evidence for causal genes in cognition. These analyses provide corroborative evidence for the causal role of NREM-skyblue in NDAD and equivocal evidence for NREM-lightyellow.

## Discussion

Alzheimer’s disease progresses over the course of many years, culminating in pathologic changes to memory, executive function, behavior, and personality. Our analyses leverage insights from genetic, transcriptional, neurophysiologic, and histopathologic data from human and mouse cohorts to investigate the common transcriptional elements shared between AD progression and NREM delta power. These experiments were designed as a molecular corroboration of a recent study that showed reduced NREM slow-wave activity in predominantly cognitive normal patients with tauopathy. We showed that two gene networks, including a CLOCK-dependent circadian network, are positively correlated with NREM delta power in a population of (C57BL/6J × 129S1/SvImJ) F2 mice and negatively correlated with tauopathy—estimated by neuropathologic measures of the Braak score and neurofibrillary tangle density—in a human AD population modeling AD progression. We confirmed that these two gene networks are particularly relevant for preclinical AD by demonstrating that they are strongly modulated in non-demented patients with neuropathologic features of AD. Lastly, probabilistic causal modeling identified *Arc, Egr2,* and *Mrpl55* as key drivers of the network associated with preclinical AD, and further analysis confirmed that both *Arc-* and *Egr2-*related subnetworks include genes that alter memory and learning*.*

Network drivers possibly play an important role in the preclinical stages of AD. *Arc* is a critical regulator of synaptic memory^70–73^ and is required for activity-dependent generation of Aβ^74^. Genetic variants of *Arc* confer genetic susceptibility to AD in Han Chinese^75^ and neuroprotection in other populations^76^. Our evidence points to its role in AD progression and NREM slow wave activity disruption. These results are consistent with our previous study, demonstrating that *Arc* is a probable upstream regulator of genes known to causally influence affective behaviors and sleep patterns^42^, two phenotypes commonly dysregulated in preclinical AD. *Egr2* also has a well-documented role in learning and memory^77^, and has also been associated with Aβ plaque-associated microglia activation^78^. In our directed networks, *Egr2* is immediately upstream of *Egr1*, a transcriptional activator of BACE-1^79^ and regulator of acetylcholinesterase^80^. *Egr2* is also binds to and transcriptionally regulates *VGF*^81,82^, a potential CSF biomarker for AD progression^83^ and a recently discovered causal network mediator of Alzheimer’s disease whose overexpression decreases cortical tau^69^. Further, *Egr2,* as well as *Arc,* are strongly downregulated by medium chain triglycerides^84^, which some evidence indicates may generally improve cognition and possibly treat APOE-negative Alzheimer’s disease^85,86^. Though there is strong evidence for the mechanisms of *Arc* and *Egr2*, there is very little experimental evidence for the biological function or pathologic role of *Mrpl55*, pointing to a potential new target for future investigation.

Our approach indirectly characterizes the molecular features of the AD prodromal phase. Understanding molecular changes in preclinical AD may reveal novel aspects of pathogenesis masked by data collected in late stage cohorts and offer new therapeutic approaches. The present study had the advantage of directly validating networks associated with NREM delta power and AD progression in a cohort of non-demented individuals with AD-related pathology, a reasonable surrogate for preclinical AD. By integrating data across multiple cohorts, preclinical molecular networks can be reasonably inferred, partially mitigating the difficulty of measuring molecular changes in preclinical AD cohorts across time.

Alzheimer’s disease and sleep deprivation have a bidirectional relationship, increasing the risk of one another and likely leading to a pathologic feedback loop^19^. Psychiatric symptoms, including depression, are also associated with neuropathologic features of Alzheimer’s disease^9,87^, possibly increasing the risk of neurodegeneration^6,7^. Our systematic approach prioritized gene networks linked to tauopathy and NREM delta power, however, this analysis recovered a network that has been previously associated with sleep deprivation and major depressive disorder^42^. Interestingly, the network exhibits features of the bidirectional relationship between sleep and AD. Sleep deprivation strongly modulates this network’s expression, while its key drivers regulate genes known to causally influence sleep–wake cycles. These findings suggest that many varied features of AD emergence and progression converge onto these cortical networks, which may serve as focal points for the complex, dynamic, and intertwined symptomatology.

Our method has several limitations that should be considered as readers interpret our results. Firstly, the transcriptome measurements in the mouse and AD cohorts were derived from bulk tissue. These data are opaque to high-resolution gene expression information, including single-cell and cell-type specific variation. A notable exception in our analyses were those data generated in non-demented individuals using laser capture microdissection. These experiments targeted specific neurons and complement our analysis of bulk tissue with higher resolution data. Another limitation is that our analysis integrates data from mouse and human brain regions that are functionally homologous to a certain degree, but have important anatomical and histological differences. Cortical homology between rodents and primates is debated, and important distinctions are often made between histological and functional homology^88,89^. The convergence of results in our experiments, despite differences between species, argues obliquely for the utility of our approach, but the interpretation of our findings should include an understanding of the inherent structural and functional differences between mouse and human prefrontal cortex. Lastly, our findings reveal molecular networks consistent with features of preclinical AD, including gene targets predicted by our modeling, and these findings were indirectly corroborated by analyzing numerous independent data sets and investigating previous experimental literature. Nevertheless, careful prospective experimental study is required to dissect the functional role of key drivers in AD prodrome and pathogenesis.

## Supplementary Information

Supplementary Information.
