# Supplementary material for: NREM delta power and AD-relevant tauopathy are associated with shared cortical gene networks
Source: Sci Rep. 2021 Apr 8;11:7797. doi: 10.1038/s41598-021-86255-6 (PMC8032807; doi:10.1038/s41598-021-86255-6)
Supplement: Supplementary file 1 — Supplementary Information. [file 41598_2021_86255_MOESM1_ESM.pdf]

NREM delta power and AD-relevant tauopathy are associated with shared cortical gene networks

Joseph R Scarpa[1], Peng Jiang[2], Vance D Gao[2], Martha H Vitaterna[2], Fred W Turek[2], Andrew Kasarskis[3*]

[1]Department of Anesthesiology, Weill Cornell Medicine, New York, NY 10065, USA

[2]Center for Sleep & Circadian Biology, Department of Neurobiology, Northwestern University, Evanston, IL, 60208, USA.

[3]Icahn Institute for Genomics and Multiscale Biology, Department of Genetics and Genomic Sciences, Icahn School of Medicine at Mount Sinai, New York, NY, 10029, USA.

*corresponding author: Andrew.kasarskis@mssm.edu

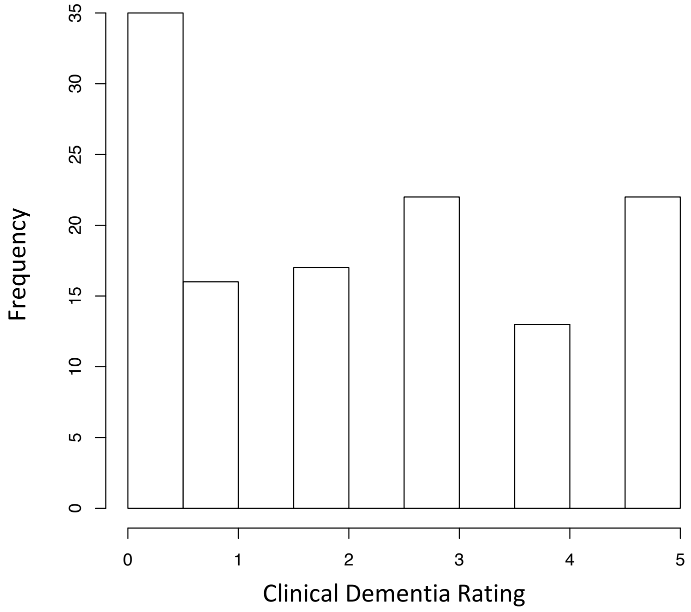

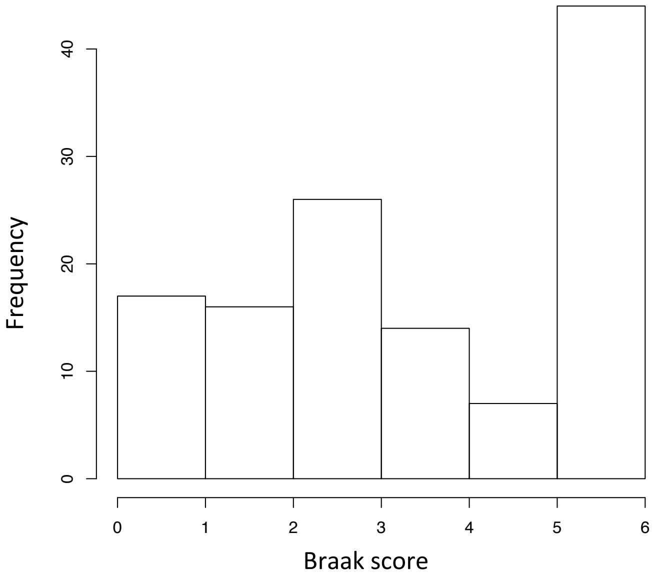

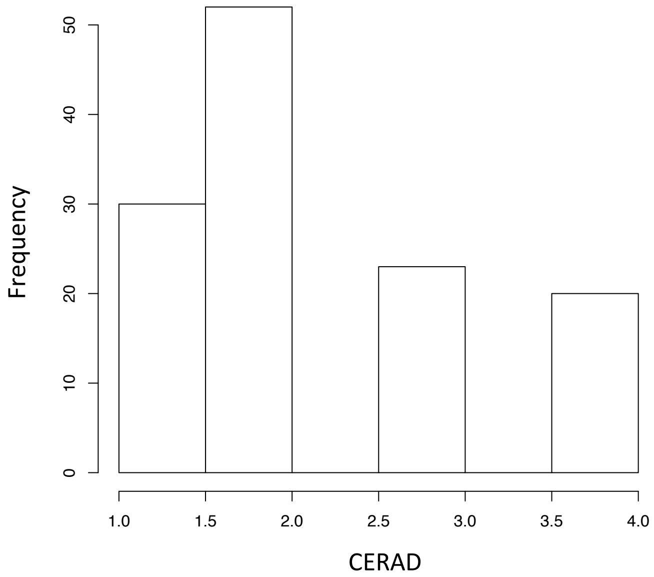

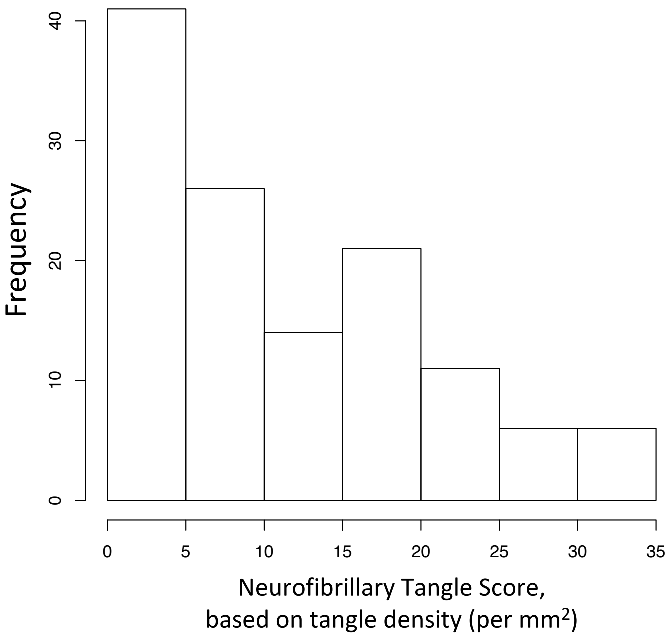

CDR	Clinical dementia rating where 0=no dementia, 0.5=questionable dementia (MCI), 1=mild dementia, 2=Moderate dementia, 3=sever dementia, 4=profound dementia, 5=terminal dementia
Braak	Braak neurofibrillary tangle score
CERAD	Neuropathology category as measured by CERAD AD diagnosis where 1=normal, 2=definite AD, 3=probable AD, 4=possible AD
NTrSum	The sum of neurofibrillary tangles (NFTs) density in hippocampus, entorhinal area, amygdala, middle frontal gyrus, superior temporal gyrus, inferior parietal cortex, and occipital cortex

Original metadata taken from Table S1 in PMID 27799057.
